# The Endocannabinoid System and Invertebrate Neurodevelopment and Regeneration

**DOI:** 10.3390/ijms22042103

**Published:** 2021-02-20

**Authors:** Tristyn L. Clarke, Rachael L. Johnson, Jonathan J. Simone, Robert L. Carlone

**Affiliations:** 1 Department of Biological Sciences, Brock University, 1812 Sir Isaac brock Way, St. Catharines, ON L2S 3A1, Canada; tc14iy@brocku.ca (T.L.C.); rj15yv@brocku.ca (R.L.J.); jsimone@brocku.ca (J.J.S.); 2 Centre for Neuroscience, Brock University, 1812 Sir Isaac brock Way, St. Catharines, ON L2S 3A1, Canada; 3 eCB Consulting Inc., P.O. Box 652, 3 Cameron St. W., Cannington, ON L2S 3A1, Canada

**Keywords:** AEA, 2-AG, CB1, CB2, endocannabinoid, regeneration, neurodevelopment, invertebrate

## Abstract

Cannabis has long been used for its medicinal and psychoactive properties. With the relatively new adoption of formal medicinal cannabis regulations worldwide, the study of cannabinoids, both endogenous and exogenous, has similarly flourished in more recent decades. In particular, research investigating the role of cannabinoids in regeneration and neurodevelopment has yielded promising results in vertebrate models. However, regeneration-competent vertebrates are few, whereas a myriad of invertebrate species have been established as superb models for regeneration. As such, this review aims to provide a comprehensive summary of the endocannabinoid system, with a focus on current advances in the area of endocannabinoid system contributions to invertebrate neurodevelopment and regeneration.

## 1. Historical Introduction to Cannabis and Endocannabinoids

Global interest in the investigation of cannabis, cannabinoids, and the endocannabinoid system has burgeoned in recent years, with numerous countries around the world introducing regulations allowing for the production of, and access to, cannabis and cannabis-derived products for medical purposes. While the adoption of formal medical cannabis regulations is relatively new in a global context, the medicinal and psychoactive properties of the plant and its derivatives have been appreciated for thousands of years.

**Figure 1:**
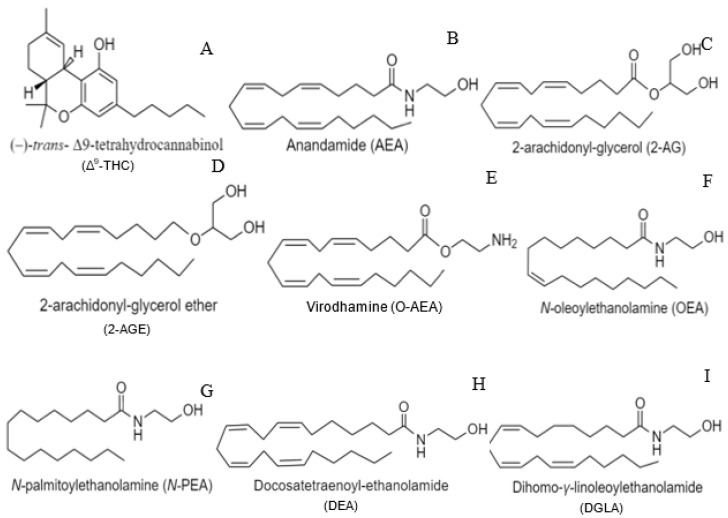
Chemical structures of various cannabinoid ligands. (**A**) Phytocannabinoid, THC. (**B**,**C**) Endocannabinoids, AEA and 2-AG. (**D**–**I**) Synthetic cannabinoids, 2-AGE, O-AEA, OEA, *N*-PEA, DEA, and DGLA.

Therapeutic uses of *Cannabis* were first described in the Pen Ts’ao Ching, the world’s oldest pharmacopoeia, for treatment of rheumatic pain, female reproductive disorders, and, when taken with wine, as an anesthetic during surgical procedures [1]. In the 1930s, (–)*trans*-Δ9-tetrahydrocannabinol (Δ^9^-THC) was identified as the major psychoactive compound in *Cannabis sativa L.*, the structure of which, however, was not determined until nearly 30 years later (Figure 1A) [2,3,4,5,6,7,8]. Initially, the lipophilicity of Δ^9^-THC was thought to non-specifically disrupt cell membranes, thus inducing its effects independent of receptor interactions [9]. However, further studies established the presence of specific inhibitory G protein-coupled receptors (GPCRs), the cannabinoid receptor type-1 (CB1) and type-2 (CB2), through which Δ^9^-THC exerts its effects [2,10,11,12]. Subsequent explorations into the physiological roles of these receptors determined that they bind with varying affinity to a number of endogenous lipid ligands (endocannabinoids), most notably *N*-arachidonoylethanolamide (AEA or anandamide) and 2-arachidonyl-glycerol (2-AG) (Figure 1B,C) [13,14,15,16,17]. While most research into endocannabinoid signaling has focused on the contributions of AEA and 2-AG, several other compounds have been identified as endocannabinoids or endocannabinoid-like molecules, including 2-arachidonyl-glyceryl ether (2-AGE), virodhamine (O-AEA), *N*-Oleoyl ethanolamine (OEA), *N*-palmitoyl ethanolamine (*N*-PEA), docosatetraenoyl-ethanolamide (DEA), and dihomo-γ-linolenoyl ethanolamide (DGLA) (Figure 1D–I) [15,16,18,19,20,21,22]. While our understanding of endocannabinoid signaling is incomplete, the diversity in known signaling compounds and modulators underscores the complexity of the endocannabinoid system and supports its role in a wide range of physiological processes.

## 2. The Endocannabinoid System in Vertebrates

### 2.1. Endocannabinoid Metabolism

**Figure 2:**
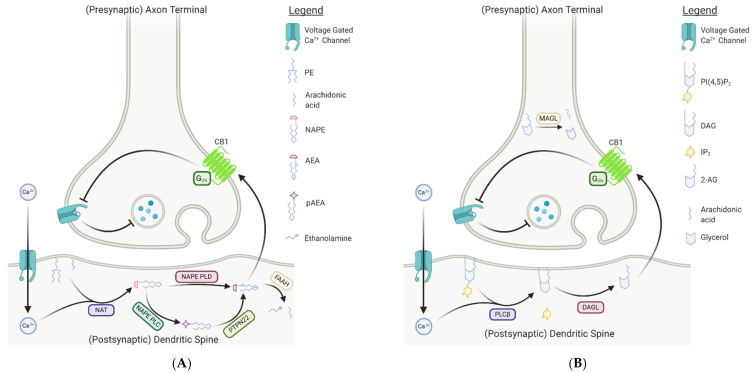
Simplified mechanism of the classical endocannabinoid ligand signaling pathway. (**A**) AEA biosynthesis and mechanism of action. In the post-synaptic neuron, NAT generates NAPE from arachidonic acid and phosphatidylethanolamine (PE) due to a calcium influx following a depolarization event. The most common pathway involves the production of AEA from NAPE through the enzyme NAPE-PLD. However, in the alternative pathway, NAPE-PLC cleaves NAPE to produce pAEA, which is rapidly dephosphorylated via PTPN22 to liberate AEA. NAPE-PLC is the exclusive AEA synthesis pathway in macrophages, key players involved in CNS regeneration. The lipophilic endocannabinoid then crosses the synapse via an unknown mechanism to interact with CB1 receptors on the presynaptic neuron. Activation of CB1 results in inhibition of neurotransmitter release through direct inhibition of voltage gated Ca^2+^ channels. AEA is catabolized in the post-synaptic neuron by FAAH to produce AA and ethanolamine. (**B**) 2-AG biosynthesis and mechanism of action. Following depolarization of the post-synaptic neuron, a calcium influx results in activated PLCβ which cleaves phosphatidylinositol 4,5-bisphosphate (PIP_2_) to produce both inositol triphosphate (IP_3_) and diacylglycerol (DAG). Subsequently, DAG is hydrolyzed by DAG lipase (DAGL), yielding 2-arachidonyl-glycerol (2-AG). The endocannabinoid then crosses the synapse similarly to AEA to bind to CB1 receptors on the presynaptic neuron. As with AEA, activation of the receptor leads to indirect inhibition of neurotransmitter release. Catabolism of 2-AG occurs in the presynaptic neuron, where the enzyme, monoacylglycerol lipase (MAGL), cleaves 2-AG into glycerol and arachidonic acid (AA). [Created with Bio-Render, BioRender.com.]

Endocannabinoids exert their neuromodulatory effects through retrograde inhibition of synaptic release via interactions with CB1 and CB2 receptors. Unlike traditional neurotransmitters and neuromodulators, endocannabinoids are poorly water soluble and thus are generally synthesized coupled to the membrane [23]. Synthesis of AEA occurs in the post-synaptic neuron, primarily through the hydrolysis of the membrane phospholipid precursor *N*-arachidonoyl phosphatidylethanolamine (NAPE) by *N*-arachidonoyl phosphatidylethanolamine-specific phospholipase D (NAPE-PLD); NAPE is generated from the membrane phospholipids phosphatidylethanolamine (PE) and arachidonic acid via an unspecified *N*-acyltransferase (NAT) [24,25]. Hydrolysis of NAPE by NAPE-PLD leads to the production of a wide variety of *N*-acylethanolamines (NAEs), including AEA (Figure 2A), and is regulated by a variety of upstream factors, including activation of ionotropic glutamate *N*-methyl-D-aspartate (NMDA) receptors, and post-synaptic responses to calcium, dopamine, glutamine, and acetylcholine [24,26,27,28,29]. While NAPE-PLD is considered the dominant synthesis pathway for AEA, enzymatic conversion of NAPE to AEA has been observed in NAPE-PLD^-/-^ mice, suggesting that alternative biosynthetic pathways likely exist [30,31]. Indeed, two additional pathways of AEA biosynthesis were subsequently discovered [24,30,31,32]. In one pathway, an alternative phospholipase, NAPE-PLC, converts NAPE to phospho-anandamide (pAEA), which is then quickly dephosphorylated by the protein tyrosine phosphatase non-receptor-type 22 (PTPN22) to free anandamide [24]. The second pathway catalyzes either NAPE or lyso-NAPE via the αβ-hydrolase domain-containing protein 4 (Abhd4) to generate the precursor, glycerophospho-arachidonoyl ethanolamide (GpAEA) [32,33]. GpAEA is then converted to AEA via the actions of the metal-dependent phosphodiesterase glycerophosphodiesterphosphodiesterase 1 (GDE1) [32,33]. While the synthesis of AEA occurs via multiple pathways, hydrolysis appears to be predominantly mediated by fatty acid amide hydrolase (FAAH) [34,35,36]. In particular, FAAH-induced hydrolysis of AEA occurs in the post-synaptic neuron and results in the production of arachidonic acid and ethanolamine [34,35,36]. Notably, FAAH was also found to catabolize 2-AG in porcine neural tissue [37]. Nevertheless, the enzymatic machinery regulating 2-AG metabolism is largely distinct from that of AEA.

The synthesis of 2-AG is achieved predominantly through the actions of post-synaptic phospholipase C-β (PLCβ). In response to a depolarization event in the post-synaptic cell, increases in intracellular Ca^2+^ drive PLCβ activity, facilitating the cleavage of phosphatidylinositol 4,5-bisphosphate (PIP_2_) into inositol triphosphate (IP_3_) and diacylglycerol (DAG) (Figure 2B) [38,39]. DAG is subsequently hydrolyzed by the enzyme diacylglycerol lipase (DAGL) to generate 2-AG [39]. The DAGL pathway is considered the dominant synthesis route for 2-AG, supported by results from DAGL knockout studies in mice, wherein a reduction in 2-AG concentrations of up to 80% in the brain and spinal cord was observed in mice lacking active DAGL [40,41]. Nevertheless, 2-AG can be generated in an alternative pathway in which PIP_2_ is dephosphorylated by PIP_2_ phosphatase producing a *sn*1-ester intermediate [42]. Hydrolysis of the intermediate via phospholipase A_1_ produces 2-arachidonoyl-lysophosphatidylinositol (LPI), which is subsequently dephosphorylated by lysophospholipase C to produce 2-AG [42,43]. Hydrolysis of 2-AG is mediated primarily through the actions of presynaptic monoacylglycerol lipase (MAGL), leading to the production of arachidonic acid and glycerol [44,45,46]. While the metabolic pathways regulating endocannabinoid synthesis and hydrolysis appear distinct, both AEA and 2-AG exert their effects through interactions with the cannabinoid receptors. Nevertheless, the spatial distribution of their respective metabolic enzymes suggests differences in their physiological contributions, with AEA likely acting as a tonic autocrine signal and 2-AG as a phasic retrograde signal.

### 2.2. The Endocannabinoid Receptors

A great deal of what we know regarding the endocannabinoid receptors derives from studies on vertebrates. In humans, the CB1 receptor consists of 472 amino acids encoded by the *CNR1* gene, sharing approximately 97% sequence identity with those of mice and rats [11,47,48]. CB1 receptors are expressed primarily in the central nervous system and are considered one of the most abundant GPCRs in the brain [11,48,49,50,51]. In addition to the canonical long form, recent research has demonstrated alternative splicing of the *CNR1* gene, resulting in two isoforms, CB1a and CB1b, each with a shorter N terminus [48]. Characterization of the expression patterns of these three isoforms has shown that although the full-length CB1 is expressed in non-neural tissues such as skeletal muscle, it remains the dominant isoform in the brain, responsible for the behavioral and psychotropic effects evoked by cannabinoids [47,48,49,50,51]. Isoform CB1b, which contains a 33 amino acid deletion at the N-terminus, showed higher expression in pancreatic β-islet cells and the liver, implicating its involvement in hepatic metabolism [48,51]. Expression of all three isoforms has also been described at low levels in Leydig cells, spermatocytes, and spermatids in the adult human, suggesting involvement in male reproductive physiology and spermatogenesis [52]. Likewise, CB1 receptors are thought to contribute to female reproductive events such as oocyte maturation, oviductal embryo transport, preimplantation embryo development, embryo implantation, placentation and even parturition [48,53]. Further, CB1 expression has also been reported in bone, skin, eyes, adipose tissue, heart tissue, the gastrointestinal tract (both in the enteric nervous system and non-neuronal cells of the intestinal mucosa), and various cancer cell types, supporting the promiscuous role of the endocannabinoid system in vertebrate physiology [48,54].

Although less characterized, CB2 receptor expression has also been reported in the skin, gastrointestinal tract, liver, and heart tissues, with predominant expression occurring on peripheral immune cells [54]. There is growing evidence supporting the presence of CB2 receptors in neural tissue. However, the precise contributions of neuronal CB2 receptors remain unclear [55,56,57]. Overall, the full-length CB2 receptor consists of 360 amino acids with only 44% homology with CB1 in humans and is comparatively less conserved among other species [48]. The CB2 receptor is encoded by the *CNR2* gene in humans, polymorphisms of which have been associated with depression, eating disorders, substance abuse, neuroinflammation linked to traumatic brain injury, multiple sclerosis, HIV-induced encephalitis, and in Huntington’s disease, Parkinson’s diseases and Alzheimer’s disease in human populations [58,59,60]. As with CB1, two isoforms of the CB2 receptor have been identified in humans [48,60]. One isoform, CB2b, is expressed principally in the spleen and other peripheral tissues, consistent with previous observations [48,60]. The other isoform, CB2a, is expressed in the testis and to a lesser extent in the reward regions of the brain, where it is believed that ligand binding exerts positive reinforcing effects by increasing dopaminergic transmission [47,48,60,61,62].

While current models of endocannabinoid signaling often focus only on contributions of CB1 and CB2 receptors, it is worth noting that both AEA and 2-AG have been reported to interact with various non-CB receptors, such as the G protein-coupled receptors (GPCR) GPR18, recently characterized as the resolvin D2 (RvD2) receptor [63], and GPR55 [12,48,64]. Likewise, AEA was also found to interact with the transient receptor potential vanilloid 1 (TRPV1) receptor, independently inhibit L-type calcium channels and negatively regulate the biosynthesis and bioactivity of 2-AG in the striatum, further underscoring the potential of non-cannabinoid receptors to contribute to endocannabinoid signaling processes [48,54,65].

### 2.3. Endocannabinoid Regulation of Synaptic Communication

Regulation of synaptic communication is considered the canonical role of endocannabinoid signaling in the vertebrate nervous system, which occurs principally through a retrograde signaling mechanism [12,48,50]. Upon depolarization of post-synaptic neurons, increases in intracellular calcium concentrations and activation of Gq/11-coupled receptors lead to the on-demand synthesis of 2-AG and AEA from lipid precursors in the post-synaptic cell membrane [12,48,50,66]. Once synthesized, endocannabinoids move retrogradely across the synapse to bind presynaptic cannabinoid receptors; activation of presynaptic CB receptors by endocannabinoids leads to the suppression of subsequent neurotransmitter release from the presynaptic terminal [12,48,50].

While both AEA and 2-AG exert their effects through CB1 receptors, differences in their relative concentrations and in their pharmacological properties suggest that they serve distinct functions. In the brain, basal concentrations of 2-AG are approximately 1000-fold greater than that of AEA, suggesting that 2-AG may act as the predominant neural endocannabinoid [48,67]. Further, while 2-AG binds CB1 receptors with comparatively low affinity, it exhibits full agonist properties, whereas AEA possesses moderate affinity for CB1 with only partial agonist properties, suggesting that differences in their neural expression may also be driven by differences in receptor affinity and/or efficacy [67]. In addition to differences in pharmacological properties, cellular recruitment of AEA and 2-AG has been shown to be stimulus-dependent, providing further support for the idea that AEA and 2-AG uniquely contribute to the regulation of synaptic communication [50,68].

The specific molecular mechanisms underlying CB1 receptor-induced reductions in presynaptic neurotransmitter release are multifaceted, involving direct G protein inhibition of presynaptic voltage-gated calcium channels (VGCCs), activation of G protein-coupled inwardly rectifying potassium channels (GIRKs), and the inhibition of adenylyl cyclase and subsequent increases in the activation of cyclic adenosine monophosphate (cAMP)-dependent protein kinase A (PKA) [48,50,69,70]. In addition to the classical models of CB1 receptor-mediated suppression of neurotransmitter release, growing evidence suggests that endocannabinoid signaling may also occur via non-classical mechanisms. For instance, evidence has emerged suggesting that AEA may act in an autocrine manner at post-synaptic CB1 and TRPV1 receptors [71,72,73,74]. Additionally, both astrocytes and microglia are capable of indirect suppression of pre- and post-synaptic neuronal transmission via the synthesis and release of AEA and 2-AG and subsequent interactions with neuronal CB1 receptors [48,50].

While CB1 receptors are considered the primary neuronal cannabinoid receptor responsible for endocannabinoid-mediated processes within the CNS, recent evidence has demonstrated the expression of central CB2 receptors, both on glial and neuronal cell populations [59]. Although the precise role of central CB2 receptors remains unclear, their intracellular expression in medial prefrontal cortical pyramidal neurons is thought to contribute to the opening of calcium-activated chloride channels, and subsequent reductions in neuronal firing, further highlighting the complex and nuanced influence of endocannabinoid signaling on synaptic communication [48,75].

### 2.4. Neurodevelopmental Contributions of Endocannabinoid Signaling

Over the past several decades, the endocannabinoid system has emerged as a key regulator of embryogenesis and neurodevelopment in vertebrates [48,49,60,76]. In particular, the ECS has been shown to contribute to axonal growth and development, with antagonism of CB1 receptors on developing CNS neurons inhibiting axonal growth [49,77]. Likewise, endocannabinoids have been shown to act as chemo-attractive and chemo-repulsive agents, shaping the connectivity of local GABAergic interneurons in the developing cerebrum [49,78,79,80]. Further, studies have implicated a role of endocannabinoids in axonal growth, fasciculation and elongation as well as proliferation and differentiation of neural progenitor cells in both mammalian and non-mammalian vertebrates, highlighting ECS-mediated contributions to CNS development [49,81,82,83,84,85]. In addition to roles in axonal growth and development, there is an emerging consensus that endocannabinoid signaling is integrally involved in adult neurogenesis: neural stem cell proliferation has been shown to be substantially reduced in both the hippocampus and the subventricular zone (SVZ) following inhibition of CB1 and/or CB2 via selective antagonists [81,82,84]. The involvement of the ECS in neurogenesis has led to a particular interest in its role in cellular migration following CNS injury [86]. Migration of neuroblasts from the SVZ of the hippocampus and the rostral migratory stream of the adult mouse brain is thought to be critical for the mitigation of injury and restoration of function in damaged brains; antagonists for CB1 and CB2 resulted in up to an 80% inhibition in neuroblast migration [86]. Endocannabinoid signaling increases during brain injury and has the capability to cross-talk with many molecules regulating neuroblast migration, such as neural cell adhesion molecules, matrix metalloproteinases, the ephrin family of receptor tyrosine kinases, and various growth factors, allowing for the integration of several common signaling pathways to produce migratory responses [86]. Likewise, proliferation and migration of a neural stem cell line to a wound site involves 2-AG signaling, altogether supporting a role of the endocannabinoid system in neurogenesis [86].

In addition to endocannabinoid involvement in neurogenesis, CB1 signaling is also involved in the promotion of cell survival or death via the activation of several mitogen-activated protein kinase (MAPK) pathways, most notably the extracellular signal regulated kinase-1 and -2 (ERK1/2) pathways, and the phosphoinositide 3-kinase (PI3K)/protein kinase B (Akt) (PI3K/Akt) pathway; the outcome of CB1-mediated signaling (i.e., cell survival or cell death) appears to be dependent on the ligand and the subcellular environment [48]. The PI3K/Akt pathway is a key regulator of cell growth and death and has been shown in several human and rat cell lines to be activated by CB1; CB1-induced activation of the PI3K/Akt pathway has been found to promote cell survival via protection against neurotoxins, trophic-deprivation, and excitotoxicity [48,87,88,89,90,91]. In the context of excitotoxicity, CB1 activation leads to PI3K/Akt -mediated increases in the expression of brain-derived neurotrophic factor (BDNF) [48,92]. BDNF, acting through a series of second messengers, activates the transcription factor NF-kB, inducing the expression of several antioxidant enzymes and anti-apoptotic proteins and providing protection against excitotoxicity [93].

### 2.5. Endocannabinoid Regulation of Immune Responses

**Figure 3:**
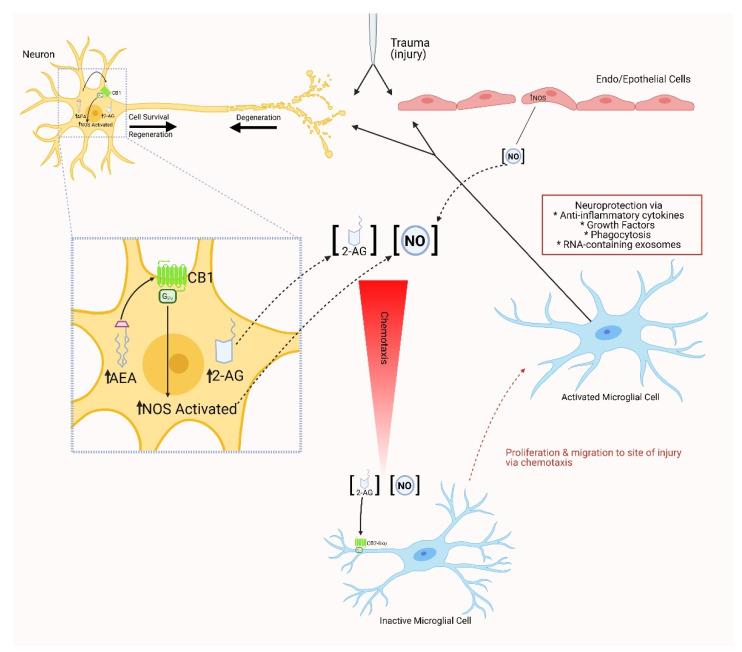
Experimental model summarizing the role of endocannabinoid signaling and nitric oxide production in the activation and chemotaxis of microglia after nervous system injury in the leech. CNS injury elicits an increase in nitric oxide synthase activity in both endo- and epithelial cells, as well as damaged neurons. NO diffuses away from the injury site to promote the concentration-dependent directed movement of activated microglia to the damaged area. At the same time, activation of anabolic enzymes in damaged neurons leads to the increased production of the endocannabinoids AEA and 2-AG. AEA can act in an autocrine manner through CB1-like receptors on neurons to increase the production of NO at the lesion site as well as promote neurite growth. 2-AG, at the same time, acts as a chemoattractant for microglia via CB2-like receptors on the lamellipodia of these immunomodulatory cells. Activated microglia accumulate at the lesion site and provide neuroprotection with the release of anti-inflammatory cytokines, growth factors and RNA-containing exosomes as well as phagocytic activity to clean up cellular debris. (With contributions from [101,102]. [Created with Bio-render, BioRender.com (accessed on 21 January 2021).]

In addition to its role in regulation of synaptic communication and neurodevelopment, the ECS also plays a critical role in the regulation of immune function, particularly following CNS injury. A simple model outlining the roles of endocannabinoid signaling and nitric oxide in microglial activation and chemotaxis after nervous system injury in the leech, is provided (see Figure 3). Of note, however, the immunomodulatory effects of endocannabinoid signaling in response to stress factors associated with neurodegeneration and CNS injury appear less dependent on CB1 receptor contributions. In fact, results from some studies suggest that endocannabinoids, acting through CB1 receptors, may actually promote tissue injury and neurodegeneration [94]. Further, the CB1 receptor antagonist/reverse agonist rimonabant has been reported to have neuroprotective effects, abolishing the long-term increase in seizure susceptibility following head trauma in rats [95], an effect that may be explained by a shift in endocannabinoid signaling away from CB1 and towards CB2 receptors.

Indeed, much of the recent literature has centered on the role of the CB2 receptor in modulating the inflammatory responses that contribute to the pathogenesis associated with neurodegenerative disease or following CNS injury. Indeed, CB2 activation in microglia and other invasive immune cells is associated with a significant reduction in excitotoxicity, apoptosis and oxidative stress [96,97,98]. Changes in CB2 receptor expression and endocannabinoid levels have been reported in almost all neurodegenerative diseases and after CNS trauma [98]. As a consequence, numerous therapeutic strategies aimed at inhibiting the enzymes associated with endocannabinoid breakdown such as MAGL and FAAH, thus increasing the abundance of 2-AG and AEA respectively, have been utilized with mixed results [96].

In addition to regulation of cell death and survival, CB1-mediated activation of the PI3K/Akt and ERK1/2 pathways is thought to underlie, in part, the effects of cannabinoids on oocyte maturation and embryonic development [53]. Specifically, CB1 receptor signaling during in vitro oocyte maturation resulted in the modified phosphorylation status of Akt and ERK1/2, both of which are involved in the regulation of spindle organization and function, polar body emission, and pronucleus formation [53,99,100]. Of note, in the absence of CB1 receptors, both in vitro and in vivo embryo development were impaired, providing support for the hypothesis that CB1 receptors play a key role in vertebrate reproductive development via their influence on intracellular signaling processes, such as the PI3K/Akt and ERK1/2 pathways [53].

## 3. The Role of the Endocannabinoid System in Invertebrates

The endocannabinoid system is phylogenetically ancient, but its presence and function in invertebrate systems have only recently been the subject of investigation [103]. CB1/CB2-type receptor-encoding genes were previously thought to be found only in those invertebrate groups most closely related to the vertebrates, specifically the phylum Chordata [104,105,106]. Recent studies, however, have provided evidence supporting a role for the ECS in numerous invertebrate phyla [36,103,104,105,107,108,109]. While the presence of cannabinoid receptors is still under investigation for many invertebrate phyla, the enzymes involved in endocannabinoid biosynthesis and inactivation/catabolism occur in most invertebrates, further supporting the existence of an endocannabinoid-like system in invertebrates [103,107,110]. A brief overview of the endocannabinoid system components and function in various invertebrate phyla follows (see Table 1).

### 3.1. Porifera

Although cannabinoid binding is yet to be observed in the sponges, and Porifera lack any form of nervous system, a phosphate-containing steroid, desulfohaplosamate, has been isolated from *Dasychalina* sp. which exhibits selective affinity for mammalian CB2 receptors [111]. Not only is this the first example of a component of an endocannabinoid system in the phylum, haplosamate derivatives are also the first cannabinoid agonists belonging to the class of steroids [111]. Furthermore, a semi-synthetic analogue of desulfohaplosamate with a cleaved ring B presented a complete loss of affinity for either CB1 or CB2, thereby demonstrating the significance of an intact steroid nucleus for haplosamate binding to these receptors [111].

### 3.2. Cnidarians

The phylogenetically oldest organism with a putative endocannabinoid system is *Hydra*, which is also considered to have one of the most primitive nervous systems [112]. Although *Hydra* do not have verified CB1 or CB2 genes, previous studies have demonstrated the presence of 2-AG, anandamide and the enzymes involved in the biosynthesis and catabolism of anandamide, NAPE and FAAH, respectively, in the membranes of *Hydra vulgaris* polyps [112]. Selective cannabinoid binding sites that behave similarly to those of CB1 and a putative role for this system in feeding behavior have also been revealed [112]. Following exposure to exogenous AEA, *Hydra vulgaris* exhibited an accelerated mouth closure event, inhibiting the cnidarian’s glutathione-induced feeding response [112]. Results were maximal at concentrations of 100 nM and reversable in the presence of the selective antagonist/inverse agonist, SR141716A [112].

### 3.3. Arthropoda

The dearth of classical endocannabinoids in the arthropod phylum, nd the lower concentrations of 2-AG in those that do produce the lipid, may be reflective of the paucity of the 2-AG precursor, esterified arachidonic acid, in arthropod phospholipids compared with mammals and other invertebrates [46]. Notably, minute measurable amounts of 2-AG and an anandamide congener, *N*-palmitoyl ethanolamine (*N*-PEA), have been isolated from neural tissues of both the fruit fly (*Drosophila melanogaster*) and the honeybee (*Apis mellifera*), as well as the salivary glands of the lone star tick (*Amblyomma americanum*) [113,114] The presence of 2-AG, *N*-PEA, and other NAEs in the obligatory ectoparasite, *A. americanum,* suggests a possible role for endocannabinoids in the inhibition of host defense reactions [114]. Similarly, the defense glands of *Agabus affinis*, an aquatic beetle, has been demonstrated to contain 2-AG [115].

Further research on *D. melanogaster* has resulted in the discovery of a DAGL ortholog (dDAGL) and a putative non-FAAH amidase, although the involvement of the latter in endocannabinoid signaling has yet to be demonstrated [113,116]. Recently it was discovered that dDAGL produces the main endocannabinoid in the adult fly, 2-linoleoyl-glycerol (2-LG), a 2-acyl glycerol that was demonstrated to bind to human CB1 receptors expressed in cholinergic neurons of genetically modified *D. melanogaster* [116]. This binding of 2-LG to the human CB1 receptors resulted in a signal cascade through the ERK and Akt kinase pathways, similar to that of their mammalian counterparts, leading to impaired motor coordination [116]. dDAGL is dynamically expressed in the fly’s brain and nerve cord during larval development, colocalizing with neuronal markers as also seen in vertebrates [116]. Expression of a mutant dDAGL, lacking catalytic activity, in *D. melanogaster* resulted in impaired axonal growth and guidance, thus causing defects in muscle innervation [116]. The specific receptor mediating these effects has not been identified. Of note, a recent study has provided evidence that *D. melanogaster* respond to vaporized cannabinoids (*Cannabis sativa*) as seen through altered cardiac rhythms [117]. Although this response is likely mediated by cannabinoid-like receptors, none have yet been identified [113,116,117].

### 3.4. Echinodermata

In a pilot study, [14C]ethanolamine radiolabelling experiments have shown that significant levels of radioactivity are incorporated into a lipid with anandamide-like chromatographic behavior in the sexually mature ovaries of *Paracentrotus lividus* and *Arbacae lixula* [118]. Further investigation of *P. lividus* ovarian lipid extracts confirmed the presence of measurable, albeit low, amounts of anandamide, as well as *N*-PEA, SEA, and lipid components with the same chromatographic behavior as NAPE [118]. Whole homogenates and eggs from *P. lividus* exhibited FAAH activity capable of converting synthetic [3H]NAPE into [3H]anandamide, suggesting the presence of NAPE-PLD during development [118]. Amidohydrolase activity catalyzing the hydrolysis of anandamide and *N*-PEA to ethanolamine was also observed in whole homogenates, with similar subcellular distribution, sensitivity to inhibitors, and pH/temperature dependency profiles to those described in mammalian tissues [118].

Endogenous AEA has also been quantified in *Lytechinus variegatus* sea urchin embryos at the 8–16 cell and mid-blastula 2 stages [119]. However, ‘perturb-and-rescue’ experiments in *L. variegatus,* other sea urchin (*Strongylocentrotus droebachinesis, Strongylocentrotus purpuratus, Dendraster excenticus*) and starfish (*Pisaterochraceus*) embryos illustrate teratogenic actions of AEA in early development [119]. Furthermore, in *S. purpuratus*, pre-treatment of sperm with anandamide resulted in a concentration-dependent inhibition of fertilization through blockage of the acrosome reaction, whereas anandamide injected ova exhibited no change in receptivity of sperm [120]. While specific cannabinoid receptor(s) for AEA in sea urchins have yet to be identified, the ligand appears to function to prevent polyspermy and contribute to early embryogenesis, as AEA addition to developing sea urchin embryos also blocks the transition from the blastula to the gastrula stage but shows no effect on cleavage [120].

### 3.5. Platyhelminthes

The presence of a cannabinoid system has been demonstrated in the primitive central nervous system of *Dugesia dorotocephala,* including the presence of AEA, 2-AG, and an entourage of NAE compounds such as *N*-PEA, stearoylethanolamide (18:0 NAE; SEA), linoleoyl ethanolamine (18:2 NAE; LEA) and OEA, though 2-AG is considerably more abundant [121]. In silico analysis of the genome of *Schmidtea mediterranea* revealed putative TRPA1, TRPV-type and TRPM-type channels (orthologs for other receptors that have known interactions with cannabinoids in vertebrate systems), as well as FAAH and MAGL-like lipases [121]. Further mining of the *Schmidtea mediterranea* genome has identified two orphan GPCRs, GPR025 and GPR484 with homology to classical CB1 [122]. GPR025 shares minor homology (~26%) with the zebrafish (*Danio rerio*) CB1-like receptor and contains 10 of 13 amino acid sequences conserved and required for CB1/2 function [122,123]. GPR484 shared 23% homology with NPR-32 of *C. elegans*, both of which possess a minimum of 7 of the 13 conserved amino acids in mammalian CB1 [122,123].

Regardless of the absence of verified CB1/CB2 receptors in the platyhelminths, the effects of cannabinoids have been well documented, and may interact with the opioid system, suggesting some similarity to that interaction observed in mammals [124]. *Planaria* display specific and quantifiable stereotypical behaviors, including alterations in neural transmission, in response to psychoactive substances [125,126]. As such, planarians have been used in several studies of the effects of cannabinoids, cocaine, amphetamine, and opioid withdrawal [61,62,125,126,127,128]. Exposure to the synthetic CB1/CB2 agonist, WIN55212-2 (WIN55), stimulated planarian motor behavior in a dose-dependent manner that was similar to that observed following exposure to opioid agonists [61,62,125]. Co-exposure with cannabinoid or opioid receptor antagonists reversed these effects [125]. These behavioral homologies are suggestive of either functional interactions between endogenous cannabinoid and opioid systems or indirect stimulation of the endogenous opioid system in planarians by cannabinoids, similar to that seen in mammalian systems [124].

Additional actions of cannabinoids in planaria may be mediated through TRP-like receptors. Planarians display what is known as “scrunching” in response to noxious stimuli, such as low pH, elevated temperatures, or amputation [129,130]. This behavior is described as a cilia-independent alteration in oscillatory gait, which is thought to be mediated through the TRP channels [129,130]. As mentioned, TRPV channel activation has been observed in response to anandamide in mammalian models [131,132]. A recent study demonstrated that two previously identified TRPV-encoding genes (*DjTRPVa* and *DjTRPVb*) partially mediate anandamide sensing in *Dugesia japonica* [130]. Exposure to 100 μM of anandamide resulted in the scrunching behavior associated with noxious stimuli, as well as an unexpected increase in head lifting or head wiggling [130]. When TRPV channels, *DjTRPVa* and *DjTRPVb* were knocked down via RNA interference, a decreased behavioral response associated with a delay in onset of scrunching was observed, highlighting the involvement of TRPV channels in anandamide-induced scrunching, but also supporting the involvement of other receptor(s) in anandamide sensing [130]. In addition to these behaviors, we have recently demonstrated that anterior segments of bisected *Dugesia dorotocephala* (formerly *Dugesia japonica*) exhibited difficulty/complete inability to right when cultured with concentrations as low as 0.5 µM of the CB1 inverse agonist AM251 (1-(2,4-dichlorophenyl)-5-(4-iodophenyl)-4-methyl-N-1-piperidinyl-1H-pyrazole-3-carboxamide), suggesting that CB1 activation may contribute to coordination of muscle contractions [122].

### 3.6. Mollusca

The first demonstration of an endogenous cannabinoid system in invertebrates was shown in *Aplysia* [99,109,133]. Consistent with mammalian models, isolated *Aplysia* buccal and parieto-visceral ganglia showed depression in nerve cell excitability in response to the CB receptor partial agonist Δ^9^-THC [109,134,135]. The ganglia of this mollusc have been shown to contain measurable levels of anandamide, 2-AG, and NAPE [109,134,135]. Homologs for AEA have also been found in the mussel *Mytilus galloprovincialis,* the clam *Tapes decussatus*, and the oyster *Crassostrea* sp., similarly to the anandamide congener *N*-PEA (NAE 16:0), which has also been identified in the clams *Venus verrucose,* and *Callista chione* [134,136]. Notably, levels of NAE and its precursor, NAPE, were considerably increased in the mussel *M. galloprovincialis* 24 h post-mortem, an effect that may share similar mechanisms with the observed increase in NAE biosynthesis following cell injury [136,137]. In accordance with the presence of endocannabinoid compounds, a putative functional ortholog for the mammalian FAAH enzyme was identified in *M. galloprovincialis,* while MAGL cDNA was isolated from *Mizuhopecte yessoensis, Lottia gigantea,* and *Pomacea canaliculata* [136,138].

Saturation binding experiments utilizing the CB1 agonist [3H]CP-55,940 confirmed the presence of cannabinoid binding sites in membrane preparations of de-sheathed central ganglia complexes isolated from the adult snail, *Helix locorum* [135]. Compared to mammalian (rat) brains, *Helix* receptors were described as being in “a modest amount” in the nervous system and exhibited low affinity to CP-55,940 [135]. Western blot analysis of snail ganglia exhibited a single 63-64 kDA band, corresponding to the expected molecular weight of glycosylated mammalian CB1, further confirming the presence of CB1-like receptors in the central nervous system of *Helix* [135].

Immunostaining against the third cytoplasmic domain of the human CB1 receptor showed specific staining in the neuropiles of all ganglia, with the most intensive staining observed in the neuropiles of pedal, pleural, cerebral and buccal ganglia, as seen in *Aplysia* and mammalian models [135]. Additional CB1-immunoreactive small cells were observed as singular entities in the pleural, buccal and pedal ganglia of *Helix*, while three groups of immunoreactive cells were detected in each cerebral ganglion as well as the visceral and right parietal ganglia [135]. The neurites of these immunoreactive cells were found to be projecting mainly inside the ganglia neuropile, while the cell bodies and fibers surrounded somata of giant pleural interneurons that are involved in eliciting the head and tentacle withdrawal behavior and surrounding the basal part of the cerebellar Purkinje cell body [135]. Further supporting the putative involvement of endocannabinoids in the regulation of functional activity of pleural sensory neurons is cannabinoid-dependent short-term synaptic inhibition that was observed and reversed by AM251 as well as the anandamide-occluded long-term increase in EPSP amplitude in the snail [138].

Furthermore, although a definitive endocannabinoid system has yet to be identified in the freshwater zebra mussel, *Dreissena polymorpha*, physiological responses to cannabinoids have been documented, demonstrating the presence of a potential cannabinoid receptor [139,140]. AEA has been reported to inhibit byssal attachment of zebra mussels and is a promising ecofriendly antifoulant [139]. Exposure to ∆^9^-THC (≥0.5 mg/L) for 14 days resulted in significant imbalances in the oxidative status of *D. polymorpha* bivalves, leading to an increase in protein carbonylation, lipid peroxidation and DNA damage [140].

Finally, a recent study identified mRNA transcripts that encode two putative cannabinoid receptors in the pond snail, *Lymnaea stagnalis,* LymCBR-like 1 and LymCBR-like 2, closely related to vertebrate CB1 and CB2 receptors [103]. LymCBR-like 1 mRNA was in highest concentration in the ovotestis and gut while LymCRB-like 2 transcripts are in greatest abundance in the CNS, though the expression of these receptors is widespread and also found in the buccal mass, penis, and mantle [103]. Injection of the CB1/CB2 receptor agonist WIN55,212-2 into *Lymnaea* prior to operant conditioning, resulted in impaired learning and memory, similar to that seen in vertebrates [103]. Further to this, it was also reported that injection of the CB1 antagonist/inverse agonist AM251 was able to enhance long-term memory formation and even reduce the duration of the adverse effects on learning that severe traumatic stressors induce [103]. Shell damage in *Lymnaea stagnalis* also leads to the enhancement of long-term memory formation during shell regeneration. However, whether this stressor involves the cannabinoid system remains to be examined [141].

### 3.7. Nematoda

Electrospray ionization ion-trap tandem mass spectrometry was used to demonstrate the presence of anandamide and 2-AG as endogenous products of the nematodes *Caenorhabditis elegans, Caenorhabditis briggsae and Pelodera strongyloides* [142]. Endocannabinoid production (including anandamide, 2-AG and OEA) has also been demonstrated in the parasite *Nippostrongylus brasiliensis*, at varying concentrations throughout its lifespan [143]. Within the *N. brasiliensis* genome, genes encoding *DAGL*, *NAPE*, orthologs of *FAAH-1,* proposed MAG-degradative enzymes a b hydroxylases *ABHD-12* and *ABHD-5,* and the minor 2-AG-degradative enzyme *ABHD-6* were identified [143]. Further, synthetic and degradative enzymes of the endocannabinoid system were found to be conserved in several other parasites, such as *Ancylostoma ceylanicum, Ancylostoma duodenale, Necator americanus, Ascaris suum, Ascaris lumbricoides, Toxocara canis, Strongyloides stercoralis, Strongyloides ratti,* and *Steinerema capocapsae* [143].

More recently, it has been determined that *C. elegans* also possesses CB1-like receptors [108,123,144,145]. In a study comparing residues critical for the functionality of vertebrate CB1 to other G protein-coupled receptors (GPCRs) in the *C. elegans* genome, two neuropeptide receptors (NPRs), NPR-19 and NPR-32, were shown to have conservation of the critical amino acid residues involved in endocannabinoid ligand binding [108]. A loss of function binding assay demonstrated that NPR-19 is a functional orthologue to the mammalian CB1/2, and NPR-32, a functional homologue to GPR18 and GPR55 [108]. Putative orthologs of NPR-19 have been found in additional nematodes, including *N. americanus, A. ceylanicum*, and *Wuchereria bancrofti* [143]. NPR-19 has since been shown to be a primary receptor for endocannabinoid-mediated regulation of regenerative axon navigation and activation of monoaminergic (e.g., serotonin and dopamine) signaling to modulate behaviors in *C. elegans* [108,123,144,145]. Endogenous 2-AG and AEA bind to and activate NPR-19 in *C. elegans*, activating inhibitory signaling cascades controlling both nociception and feeding [144].

Two genes, *nape-1* and *nape-2*, adjacent to one another within the *C. elegans* genome, are functional homologs of NAPE-PLD [146,147]. Both genes are expressed predominantly in the pharynx, though *nape-2* is also found in high levels in the dorsal and ventral nerve cords, and vulval muscles of hermaphrodites [146]. Functionality of *nape-1* and *nape-2* appear to be temperature-dependent, as overexpression of *nape-1* resulted in a generalized delay in development with a more severe phenotype at 25 °C, while *nape-2* overexpression conferred a partial arrest at the first larval stage of development (L1), as well as a slight increase in lifespan most noticeably at 15 °C [146]. These orthologs of NAPE-PLD result in the liberation of a variety of NAEs, including the anandamide congener eicosapentaenoyl ethanolamide (EPEA), which was demonstrated to play a role in the development and aging of *C. elegans* through a signal cascade that coordinates metabolism with nutrient status [147]. Available evidence suggests that in response to nutrient availability, the nutrient sensing target of rapamycin (TOR) pathway may control EPEA and other NAE levels; reduced NAE levels act as a metabolic signal upstream of the Foxa transcription factor PHA-4 in the dietary restriction pathway [147]. Developmental arrest induced by cholesterol starvation has also been shown to enrich endogenous production of endocannabinoids, 2-AG and anandamide [148]. These eCBs then function to enhance cholesterol trafficking efficiency and promote the use of internal reserves, thereby rescuing the developmental arrest caused by cholesterol depletion [148].

### 3.8. Annelida

In 1997 a fragment of a putative cannabinoid receptor cDNA was cloned from the medicinal leech, *Hirudo medicinalis,* which contained two highly conserved motifs sharing 58% and 80% homology with the human CB1 receptor [149]. Subsequent immunocytochemical studies have identified this receptor in the supra-esophageal ganglia of the CNS of two mature leech species (*H. medicinalis* and *Theromyzon tessulatum*), while CB2 has been observed in neurons and glial cells of the central neuropil of the ventral chain ganglia [150]. Moreover, endocannabinoids AEA and 2-AG, and anandamide-related compounds (e.g., NAPE, *N*-PEA and LEA) have also been demonstrated in the CNS of *H. medicinalis* [150]. Similar to what is seen in vertebrates, stimulation of this CB1-like receptor by anandamide leads to the inhibition of cAMP formation through activation of nitric oxide release [109,150].

Three putative leech anandamide amidases have been shown to colocalize with the CB1-like receptors in the CNS of *H. medicinalis* [150]. Although one of these proteins exhibited an apparent molecular weight similar to that expected for mammalian FAAH enzymes, the most abundant amidase in leech cytosolic fractions is distinct from mammalian FAAH, both in molecular weight and pH dependency [150]. A hydrolase assay using whole leech CNS homogenates and cytosolic fractions demonstrated insensitivity of the leech amidase to common mammalian FAAH inhibitors and degradation of both AEA and *N*-PEA, suggesting that leech anandamide amidase activity is not selective for AEA or 2-AG, and additional enzymes for the inactivation of endocannabinoids may be present in leech CNS [150].

Recently, transcripts of a putative MAGL (HirMAGL) gene in the central nervous system of *Hirudo verbena* have also been detected [138]. In this novel study, Kabeiseman et al. (2020) became the first group to characterize an invertebrate form of MAGL, which they were also able to show mimicked the expression and localization in mammalian cells, segregating to the plasma membrane when expressed in HEK93 cells. When a leech bites a host, DAG-produced MAG is discharged from the leech salivary glands and immediately injected into the host [151]. It is believed that, in this context, the function of DAGL-produced MAGs is multifaceted, and involves: (1) blocking the host’s peripheral nociception and alleviating host pain; and (2) acting in an immunosuppressive fashion to inhibit an immunocyte response to evade host immune defense [151,152,153].

Finally, depolarization-induced suppression of neuronal activity has been documented in the medicinal leech after exposure to cannabinoid compounds [154]. Bath-application of the CB1 receptor antagonist/inverse agonist AM251 to midbody ganglia of *Hirudo* inhibited LTD of the chemical T-to-S synapse during 900 s low-frequency stimulation but not at the 450 s low frequency [154]. Inhibition of 2-AG synthesis also blocked LTD elicited by 900 s low-frequency stimulation [154].

### 3.9. Chordata

Cannabinoid receptor orthologs to both the human CB1 and CB2 receptors have been identified in the cephalochordate *Branchiostoma floridae* (Amphioxus), coined BfCBR, and the urochordate *Ciona intestinalis*, known as CiCBR [104,105,106]. Though the physiological functions of BfCBR and CiCBR are still under investigation, the expression pattern of CiCBR in the cerebral ganglion of *C. intestinalis* was determined using Western blotting and immunocytochemistry [104]. The intense immunostaining varicosities of CiCBR were reflective of the CB1 immunostaining patterns seen in mammalian forebrains, indicating CiCBR may be involved in presynaptic regulation of neurotransmitter release, similarly to mammalian CB1 [155]. In fact, it has been suggested that the role of CB1 as a regulator of neurotransmitter release in mammals may have indeed originated in these chordates [155]. Further to this, CiCBR mRNA expression was found to be in greatest abundance in the cerebral ganglion, as well as in the brachial pharynx, heart, and testis of *C. intestinalis,* which is consistent with mammalian expression patterns of cannabinoid receptors [106].

Comparatively, in *C. intestinalis,* the endocannabinoids AEA and 2-AG were present in the cerebral ganglion, heart, intestine, stomach, pharynx, ovaries, and testis, with AEA produced most abundantly in cerebral ganglion cells [106]. 2-AG was detected at 5-fold greater levels and localized primarily to the stomach and intestinal cells [106]. Levels of NAPE, *N*-PEA, and an FAAH ortholog with 44% homology to human FAAH, were found throughout these tissues as well [106]. Though the main components of the endocannabinoid system are present in *C. intestinalis,* the pharmacological properties of CiCBR have yet to be determined, thus it cannot be assumed that CiCBR responds to cannabinoids [104,106,155]. Nevertheless, the colocalization of endocannabinoid biosynthetic machinery with CiCBR is strongly suggestive of a functional interaction [106].

## 4. Endocannabinoids in Invertebrate Regeneration

In addition to the above actions of endocannabinoids in invertebrates, there is also a growing body of evidence demonstrating their involvement in several models of invertebrate regeneration. This is not surprising given the evidence for the participation of the ECS in the regulation of neural stem cell proliferation, differentiation, migration, and neurite outgrowth and guidance during vertebrate development, as discussed previously.

### 4.1. Annelida

Endocannabinoid involvement in regenerative processes within *Annelida* has been documented. Increased titres of endocannabinoids were found to enhance neurite outgrowth in the regenerating central nervous system of the leech [156]. Upon further investigation, it was determined that the effects of endocannabinoids in this instance were mediated through the activation of leech TRPV channels [156]. Another study in the leech demonstrated that the resultant nitric oxide release from neuronal injury may activate local microglial cells to stimulate their expression of CB2-like receptors which bind 2-AG [157]. Lesions to the central nervous system of the medicinal leech are thought to also induce the release of AEA, which further activates nitric oxide release by binding to the TRPV1-like receptor [157]. These findings are in concert with those of Meriaux et al. (2011), in which both AEA and 2-AG were present and peaked within the first hour following nerve cord damage, suggesting a role for endocannabinoids in modulation of brain inflammation in the leech. Altogether, the concurrent release of endocannabinoids and nitric oxide is thought to promote neuronal survival by inhibiting the accumulation of microglia at the lesion site [156,157,158].

### 4.2. Nematoda

The endocannabinoid AEA has been shown to be produced around sites of injury and to act as a putative damage signal in *C. elegans* to help regulate axon regeneration and guidance [108,123]. In contrast to leeches, however, the effect of AEA in *C. elegans* in axon regeneration is inhibitory [108,123]. As mentioned previously, *C. elegans* possess a CB1 homologue, NPR-19, which binds AEA [108]. Binding of AEA to this receptor results in the antagonism of the JNK-MAPK pathway via G_o/α_-dependent signaling, in turn causing growth cone repulsion and inhibition of regeneration [108,123].

### 4.3. Platyhelminthes

As described above, the behavioral effects of cannabinoids are well established in planarians, indicating that they likely possess receptors capable of binding endocannabinoids. Endocannabinoids are also thought to contribute to regenerative processes in planarians, as the presence of the endocannabinoids AEA and 2-AG, as well as the other NAE compounds SEA and *N*-PEA, were found to increase following transverse amputation, where significant changes were seen within the initial 12 h of regeneration in the planarian *Dugesia dorotocephala* [121].

Furthermore, a recent study conducted in our lab (Clarke, 2020) has demonstrated an AM-251 concentration-dependent inhibition of head and tail regeneration in *Dugesia dorotocephala.* Concentrations as low as 0.5 µM AM251 impaired the regeneration of head structures from posterior segments of bisected planarians [122]. Surprisingly, several anterior segments disintegrated prior to regenerating any tail structures [122]. Similarly, when *intact* planarians were incubated in 0.5 µM AM251, the heads of several specimens also disintegrated [122]. The CB2 inverse agonist AM630 ([6-iodo-2-methyl-1-[2-(4-morpholinyl)ethyl]-1H-indol-3-yl](4-methoxyphenyl)-methanone) had no effect on regeneration of either anterior or posterior segments and was not toxic at those concentrations [122]. These results strongly suggest the involvement of CB1 in survival and regeneration of the central nervous system of *Dugesia dorotocephala.*

In addition, blueberry anthocyanins (ANT) have been demonstrated to have a neuroprotective effect on *Dugesia japonica* nerve cells in both intact and amputated planarians (at the post-auricle level) following damage due to perfluorooctane sulfonate (PFOS) exposure [159]. PFOS is a perfluoroalkyl substance that has been shown to cause DNA damage, morphological changes in the planarian CNS, alterations in neurotransmitter levels and changes in neural related gene expression [159]. The PFOS (5 mg/L)-exposed group(s) resulted in brains with smaller cephalic ganglia and reduced brain branches and nerve fiber density compared to controls, whereas combined PFOS and ANT (at 10 mg/L and 20 mg/L) treatment groups had brain morphological recovery that was similar to that of controls in both the intact planarians and regenerating transverse tail segments [159]. Increased concentrations of ANT led to greater recovery of brain branches and nerve fiber densities [159]. It should be noted that anthocyanins are flavonoids and a natural, water-soluble pigment found in many flowering and fruit-bearing plants [159,160]. The anthocyanins cyanidin and delphinidin are two of the most abundant anthocyanins found in both blueberries and *Cannabis sativa*, where they have been demonstrated to act as ligands with moderate affinity to human CB1 [160,161]. Whether these anthocyanins act through CB1 signaling to mediate these gene expression changes is unclear and is a question for future studies.

## 5. Conclusions and Perspectives

Endocannabinoid signaling permeates virtually every aspect of both vertebrate and invertebrate physiology. As such, it is the subject of an increasing number of studies in a variety of organisms. In particular, its role in neural development and specifically with respect to neurogenesis, neural migration, guidance, synaptogenesis and synaptic plasticity in both vertebrates and invertebrates highlights this pathway as a key target for pharmacological intervention in the treatment of CNS injury. Going forward, it will be imperative to continue to gain a thorough understanding of the potential interactions and integration between the endocannabinoids and other critical signaling pathways, such as the retinoid, Notch and Wnt pathways, that contribute to the successful regeneration of damaged neural tissue in invertebrates. These studies would serve as a prelude to similar studies on regeneration-competent vertebrates such as the zebrafish and axolotl. Preliminary studies in our laboratory have demonstrated a critical role for activation of both CB1 and CB2 receptors in non-overlapping populations of neural progenitor cells (radial glial and subependymal cells, respectively) in the regenerating caudal spinal cord of the axolotl [162]. Indeed, a more thorough understanding of the potential interactions between the ECS and neurodevelopmental signaling processes in the axolotl and other model organisms paves the way towards the development of novel pharmacological reagents and strategies for the treatment of CNS injury in those vertebrates incapable of functional regeneration.

## Figures and Tables

**Table 1:** Summary of the Endocannabinoid System in the Invertebrates.

Species		Function of Endocannabinoid System	Location and Endocannabinoid(s) Orthologs/Enzymes Isolated		Presence of CB1/CB2-Like Receptors	Other Putative Endocannabinoid Receptors	References
*Porifera*	*Dasychalina* sp.	Unknown	Unknown	Desulfohaplosamate (steroid eCB ligand)	Unidentified		Chianese et al. (2011)
*Cnidarians*	*Hydra vulgaris*	Role in feeding: presence of exogenous AEA exhibited maximal mouth closure; selective CB1 antagonist SR 141716A reversed effect	Polyps	AEA, NAPE, 2-AG, FAAH-like activity detected	Unidentified		De Petrocellis et al. (1999)
*Arthropoda*	*Drosophila melanogaster*	Putative involvement of dDAGL in axonal growth and guidance, particularly during muscle innervation	Neural tissues	2-AG, *N*-PEA, 2-LG, non-FAAH amidase, dDAGL	Unidentified		McPartland et al. (2001), Tortoriello et al. (2020)
	*Apis mellifera*	Unknown	Neural tissues	2-AG, *N*-PEA	Unidentified		McPartland et al. (2001)
	*Amblyomma americanum*	Putative role in host defense reactions	Salivary glands	2-AG, *N*-PEA	Unidentified		Fezza et al. (2003)
*Platyhelminthes*	*Dugesia dorotocephala*	Putative role in regeneration	Central nervous system	AEA, 2-AG, *N*-PEA, SEA, LEA, OEA	Unidentified		Mustonen (2010), Clarke (2020)
	*Schmidtea mediterranea*	Unknown	Genome (*in silica* analysis)	FAAH, MAGL	GPCR025 (26% homology with *Danio rerio* CB1-like receptor); GPCR484 (23% homology with NPR-32 of *C. elegans*)	TRPA1 channels, TRPV-type channels, TRPM-type channels	Mustonen (2010), Clarke (2020)
*Mollusca*	*Aplysia*	Unknown	Unspecified	AEA, 2-AG, NAPE	Unidentified		Lemak et al. (2007)
	*Mytilus galloprovincialis*	Role in injury response	Unspecified	AEA, *N*-PEA, NAPE, FAAH ortholog	Unidentified		Sepe et al. (1998)
	*Tapes decussatus*	Unknown	Unspecified	AEA, *N*-PEA	Unidentified		Sepe et al. (1998)
	*Crassostrea* sp.	Unknown	Unspecified	AEA, *N*-PEA	Unidentified		Sepe et al. (1998)
	*Venus verrucose*	Unknown	Unspecified	*N*-PEA	Unidentified		Sepe et al. (1998)
	*Callista chione*	Unknown	Unspecified	*N*-PEA	Unidentified		Sepe et al. (1998)
	*Mizuhopecte yessoensis*	Unknown	Unspecified	MAGL cDNA	Unidentified		Kabeiseman et al. (2020)
	*Lottia gigantea*	Unknown	Unspecified	MAGL cDNA	Unidentified		Kabeiseman et al. (2020)
	*Pomacea canaliculata*	Unknown	Unspecified	MAGL cDNA	Unidentified		Kabeiseman et al. (2020)
	*Helix locorum*	Unknown	Unspecified	Unspecified	CB1-like receptor		Lemak et al. (2007)
	*Dreissena polymorpha*	Unknown	Unspecified	Unspecified	Potential eCB-like receptor		Angarano et al. (2009), Parolini et al. (2014)
	*Lymnaea stagnalis*	Learning and memory; long-term memory enhanced in response to endocannabinoid antagonist			LymCBR-like 1 (transcripts in greatest abundance in ovotestis and gut); LymCBR-like 2 (transcripts in greatest abundance in central nervous system)		Sunada et al. (2017)
*Nematoda*	*Caenorhabditis elegans*	Helps regulate regenerative axon navigation and activate monoaminergic signaling; activation of inhibitory signaling cascades in nociception and feeding; development and aging	Pharynx	*nape-1* and *nape-2*	NPR-19 (functional ortholog to mammalian CB1/2 receptor); NPR-32 (functional homolog to GPR18 and GPR55)		Pastuhov et al. (2016), Oakes et al. (2017), Lucanic et al. (2011), Harrison et al. (2014)
			Dorsal and ventral nerve cords, hermaphrodite vulval muscles	*nape-2*			
			Unspecified	AEA, 2-AG			
	*Caenorhabditis briggsae*	Unknown	Unspecified	AEA, 2-AG	Unidentified		Lehtonen et al. (2008)
	*Pelodera strongyloides*	Unknown	Unspecified	AEA, 2-AG	Unidentified		Lehtonen et al. (2008)
	*Nippostrongylus brasiliensis*	Unknown	Unspecified	AEA, 2-AG, OEA, NAPE, FAAH-1-like orthologs, MAG-degradative enzymes (i.e., ABHD-12, ABHD-5, ABHD-6), DAGL-like genes	Unidentified		Batugedara et al. (2018)
	*Necator americanus*	Unknown	Unspecified	Unspecified	Putative orthologs of NPR-19		Batugedara et al. (2018)
	*Ancylostoma ceylanicum*	Unknown	Unspecified	Unspecified	Putative orthologs of NPR-19		Batugedara et al. (2018)
	*Wuchereria bancrofti*	Unknown	Unspecified	Unspecified	Putative orthologs of NPR-19		Batugedara et al. (2018)
*Annelida*	*Hirudo medicinalis*	Unknown	Central nervous system	AEA, 2-AG, NAPE, *N*-PEA, LEA	CB1-like and CB2-like receptors (transcripts isolated in supra-esophageal ganglia)		Matias et al. (2001)
	*Theromyzon tessulatum*	Unknown	Unspecified	Unspecified	CB1-like and CB2-like receptors (transcripts isolated in supra-esophageal ganglia)		Matias et al. (2001)
	*Hirudo verbana*	Immunomodulatory functions including modulation of brain inflammation; regeneration	Unspecified	AEA, 2-AG, NAPE	High affinity anandamide receptors	TRPV channels	Salzet and Stefano (2002), Meriaux et al. (2011), Arafah et al. (2013), Matias et al. (2001), Kabeiseman et al. (2020)
			Central nervous system	HirMAGL			
*Echinodermata*	*Paracentrotus lividus*	Role in development; polyspermy block	Mature ovaries	AEA, NAPE, FAAH activity	Unidentified		Bisogno et al. (1997), Buznikov et al. (2009)
*Chordata*	*Branchiostoma floridae* (Amphioxus)	Unknown	Unknown		BfCBR		Buznikov et al. (2009)
	*Ciona intestinalis*	Potential involvement in neurotransmitter release and neuromodulation	Cerebral ganglion, heart, intestine, stomach, pharynx, ovaries, testis	AEA, 2-AG, NAPE, *N*-PEA, FAAH	CiCBR (mRNA expressed in cerebral ganglion, brachial pharynx, heart and testis)		Égertova and Elphick (2007), Matias et al. (2005), Elphick et al. (2003)

## Data Availability

Not applicable.
